# Calcium sulfide nanoparticles as sustained H_2_S donors with neuroprotective potential in ischemic stroke

**DOI:** 10.3389/fimmu.2026.1746066

**Published:** 2026-04-17

**Authors:** Zhen Lu, Yu-Bin Liang, Zhaotao Wang, Jianghui Xie, Yiqing Chen, Yang Li, Zhiqiang Peng

**Affiliations:** 1 Postgraduate Cultivation Base of Guangzhou University of Chinese Medicine, Panyu Central Hospital, Guangzhou, China; 2 Department of Stroke Center, The Affiliated Panyu Central Hospital of Guangzhou Medical University, Guangzhou, China; 3 Department of Neurosurgery, The Second People’s Hospital of Fujian Province, The Second Affiliated Hospital of Fujian University of Traditional Chinese Medicine, Fuzhou, China; 4 Department of Neurosurgery, Institute of Neuroscience Key Laboratory of Neurogenetics and Channelopathies of Guangdong Province and the Ministry of Education of China, The Second Affiliated Hospital of Guangzhou Medical University, Guangzhou, China; 5 School of Biomedical Engineering, Guangzhou Medical University, Guangzhou, China

**Keywords:** anti-apoptosis, anti-inflammation, antioxidation, calcium sulfide nanoparticles, hydrogen sulfide, ischemic stroke, sustained-release donor

## Abstract

**Introduction:**

Acute ischemic stroke remains a major cause of death and disability, underscoring the need for safe and effective neuroprotective strategies. Hydrogen sulfide (H₂S) exhibits dose-dependent neuroprotective effects but its therapeutic application is constrained by volatility and burst release.

**Methods:**

We synthesized low-solubility, slowly hydrolyzing calcium sulfide nanoparticles (CaS NPs) via a wet-chemistry route as an intrinsically slow-releasing H₂S donor. Their sustained release profile was characterized, and efficacy was evaluated in vitro using SH-SY5Y cells under oxygen-glucose deprivation/reoxygenation (OGD/R) and in BV2 microglia, and in vivo using a distal middle cerebral artery occlusion (dMCAO) mouse model.

**Results:**

CaS NPs demonstrated sustained H₂S release over 48 h. In vitro, they enhanced SH-SY5Y cell viability under OGD/R, decreased intracellular reactive oxygen species, suppressed TNF-α and IL-1β expression in BV2 cells, and reduced neuronal apoptosis. In the dMCAO model, CaS NPs increased cortical H₂S levels, improved 24-h neurological scores, reduced day-3 infarct area, preserved peri-infarct neurons, mitigated ROS accumulation, and attenuated astrocyte and microglia activation. Treatment consistently decreased Bax expression, increased Bcl-2 levels, and reduced pro-inflammatory cytokine expression. Short-term safety assessments indicated a favorable biosafety profile.

**Discussion:**

Collectively, these findings provide proof-of-concept support that CaS NPs can serve as a slow-releasing H₂S donor platform for further evaluation in experimental ischemic stroke."

## Introduction

Acute ischemic stroke is a leading cause of death and long-term disability worldwide, with ischemic events accounting for approximately 80% of all strokes (1–3). Following arterial occlusion, a cascade of metabolic failure, oxidative stress, neuroinflammation, and apoptotic signaling contributes to progressive neuronal loss, particularly within the ischemic penumbra, where tissue remains potentially salvageable (4, 5). Recent nanomedicine studies further highlight that secondary brain injury after stroke involves interconnected inflammatory and oxidative pathways that may benefit from multi-target therapeutic approaches, including nanosystems designed to regulate inflammatory mediators and reactive oxygen species (6, 7). While reperfusion therapies, including intravenous thrombolysis and mechanical thrombectomy, remain the cornerstone of acute management, their benefit is constrained by narrow time windows, eligibility limits, and risks such as hemorrhagic transformation (8, 9). Consequently, there is sustained interest in adjunct neuroprotective strategies that may help stabilize the penumbral microenvironment and broaden the therapeutic window.

H_2_S has emerged as an important gaseous signaling molecule with context- and dose-dependent neuroprotective effects, including antioxidant activity, mitochondrial preservation, anti-inflammatory signaling, and anti-apoptotic regulation (10, 11). Recent studies further suggest that controlled H_2_S delivery systems may influence the post-injury microenvironment by reducing oxidative stress and inflammatory signaling and by supporting neuronal survival, thereby motivating further investigation of regulated H_2_S release in nervous system injury (12). Despite these promising biological effects, the therapeutic translation of H_2_S remains challenging because its delivery kinetics are difficult to control precisely *in vivo*. Commonly used inorganic donors such as NaHS and Na2S are highly water-soluble and rapidly release H_2_S upon dissolution, resulting in burst release, short biological half-life, and potentially toxic local concentration peaks (13, 14). Although several organic donors and hybrid carrier systems have been developed to moderate H_2_S release, these approaches may introduce additional formulation complexity, batch variability, and potential biocompatibility concerns (15). Recent advances in nanomedicine have further expanded strategies for controlled H_2_S delivery. Recent advances in nanomedicine have expanded strategies for controlled H_2_S delivery across diverse disease settings, further supporting the feasibility of nanomaterial-based platforms for regulating H_2_S release *in vivo (*16, 17).

Materials with intrinsically low aqueous solubility provide an alternative strategy for sustained H_2_S generation by functioning as solid-state reservoirs that gradually release sulfide through controlled hydrolysis. Calcium sulfide nanoparticles (CaS NPs) represent one example of this concept. Owing to their limited solubility in aqueous environments, CaS NPs undergo slow hydrolysis under physiological conditions, enabling more gradual H_2_S generation than rapidly dissolving inorganic sulfide salts. Previous studies suggest that this hydrolysis-mediated mechanism can sustain H_2_S release for more than 12 h under physiological conditions, thereby providing a prolonged exposure profile in biological systems (18). This release behavior may be relevant to ischemic stroke, in which secondary injury evolves over an extended time window characterized by persistent oxidative stress, glial activation, and delayed neuronal death. However, achieving H_2_S exposure profiles that remain compatible with this prolonged pathological timeline remains challenging, highlighting the need to evaluate delivery platforms capable of maintaining controlled H_2_S availability throughout the secondary injury phase.

Based on this rationale, we developed CaS NPs as an intrinsic, carrier-free, slow-releasing H_2_S donor candidate. Because CaS is sparingly soluble and undergoes gradual hydrolysis in aqueous media, it may provide a prolonged H_2_S release profile extending up to 48 h under the tested conditions. We hypothesized that such kinetics may better correspond to the temporal dynamics of post-ischemic injury, potentially providing early antioxidant support together with continued modulation of inflammation- and apoptosis-related processes while limiting high transient peaks. Using a wet-chemistry synthesis approach, we obtained crystalline CaS NPs with a relatively uniform size distribution and verified sustained H_2_S generation using qualitative and quantitative assays.

**Scheme 1:**
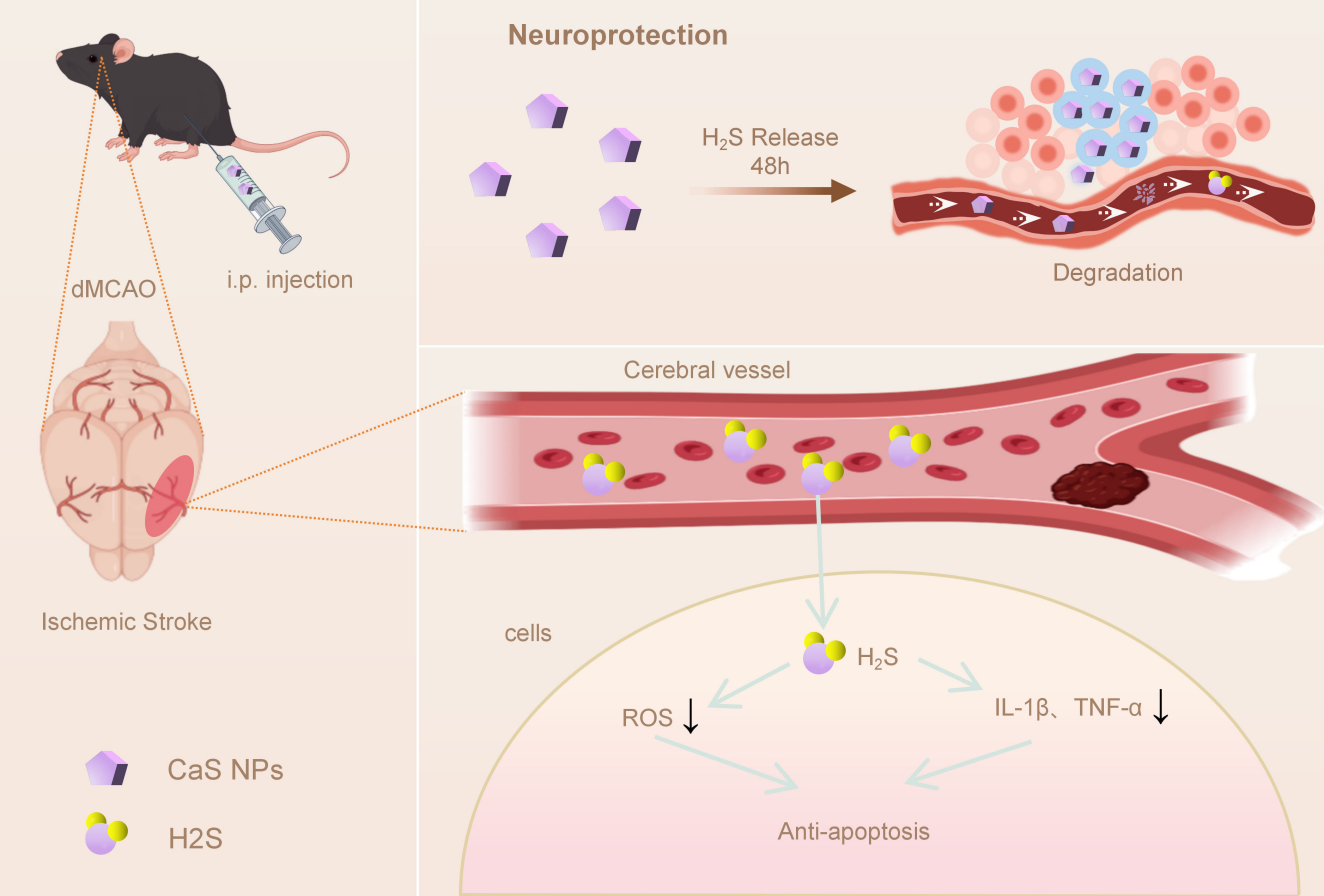
Schematic of the release process of CaS NPs and its neuroprotective effect in acute ischemic stroke.

We then evaluated the neuroprotective potential of CaS NPs in complementary *in vitro* and *in vivo* models (**Scheme 1**). *In vitro*, oxygen-glucose deprivation/reoxygenation (OGD/R) assays in SH-SY5Y neurons and BV2 microglia were used to assess cell injury-related, oxidative stress-related, inflammatory, and apoptosis-related readouts. *In vivo*, a distal middle cerebral artery occlusion (dMCAO) mouse model was used to assess whether CaS NPs were associated with improved neurological outcomes, reduced infarct development, preserved peri-infarct neurons, and attenuated glial activation, alongside preliminary short-term biosafety profiling. Overall, this study provides proof-of-concept evidence supporting further evaluation of CaS NPs as a simple controlled H_2_S donor platform for experimental ischemic stroke.

## Materials and methods

### Synthesis and characterization of CaS NPs

#### Materials

Sodium sulfide nonahydrate (Na_2_S·9H_2_O, 99.9%), calcium chloride dihydrate (CaCl_2_·2H_2_O, 99.9%), 1-thioglycerol (95%), tetrahydrofuran (99.5%), and tetraethyl orthosilicate (TEOS, >99%) were sourced from Macklin (Shanghai, China). Ammonia solution (NH_3_·H_2_O, AR) was purchased from Aladdin (Shanghai, China). All chemicals used were of analytical grade and employed without further purification, unless stated otherwise.

#### Synthesis of CaS NPs

CaS nanocrystals were synthesized via a wet chemical co-precipitation method, based on a procedure from a previous study (19). Briefly, 0.5505 g of CaCl_2_·2H_2_O was dissolved in 150 mL ethanol to make a 0.025 M solution. A 0.0125 M sulfur source solution was prepared by dissolving 0.4503 g Na_2_S·9H_2_O and 0.165 mL 1-thioglycerol in 150 mL ethanol, followed by sonication for 30 minutes. Both solutions were freshly prepared. The CaCl_2_·2H_2_O solution and varying amounts of the sulfur source solution were combined in a three-neck flask. After 15 minutes of nitrogen deoxygenation, 150 mL of Na_2_S·9H_2_O solution was injected rapidly into the mixture at room temperature while stirring vigorously for 12 hours. The nanoparticles formed were then treated with 30 mL tetrahydrofuran dropwise and stirred under a nitrogen atmosphere for 5 hours. The solution was concentrated by rotary evaporation, and the nanoparticles were isolated by centrifugation, followed by vacuum drying to remove any residual ethanol. Finally, the nanoparticles were annealed in a silica crucible at 800 °C for 2.5 hours in the presence of sulfur powder under nitrogen, and the resulting nanoparticles were collected after cooling to room temperature for further characterization.

#### Characterization

The crystallinity of the synthesized material was confirmed as pure CaS using X-ray diffraction (XRD, Bruker D8, Germany). Transmission electron microscopy (TEM, Talos F200S, FEI Thermo, Czech Republic) was used to assess the size and morphology of the nanoparticles.

#### Generation from CaS NPs

The ability of CaS NPs to generate H_2_S was visually assessed by preparing an aqueous solution of 3000 μM CaS NPs. The supernatant was applied to lead acetate test papers at different time intervals. The formation of H_2_S was indicated by blackening of the paper due to the reaction of H_2_S with lead acetate to form lead sulfide. For quantitative analysis, 300 μM aqueous solutions of CaS NPs were mixed for various durations (0–48 hours). After centrifugation, the supernatant was mixed with a micro H_2_S assay kit (G0133W, Grace Biotechnology, Suzhou, China). Absorbance at 665 nm was measured after a 15-minute incubation at 25 °C to determine the H_2_S release kinetics.

### *In vitro* experiments

#### Materials

Dulbecco’s Modified Eagle Medium (DMEM), penicillin, and streptomycin were obtained from Thermo Fisher Scientific (Massachusetts, USA). Trypsin was purchased from NCM Biotech (Suzhou, China). The ROS assay kit was obtained from Elabscience (Wuhan, China). Fetal bovine serum (FBS) and the Annexin V-7-AAD Apoptosis Detection Kit were sourced from Pricella (Wuhan, China).

#### Cell lines

Human neuroblastoma SH-SY5Y and mouse microglia BV2 cells were procured from Pricella Life Technology Co., Ltd. (Wuhan, China). Cells were cultured in DMEM (high glucose) supplemented with 10% FBS, 100 µg/mL streptomycin, and 100 U/mL penicillin. Cultures were maintained in a humidified atmosphere at 37 °C with 5% CO_2_.

*In Vitro* Cytotoxicity: To assess the biocompatibility of CaS NPs, SH-SY5Y cells were incubated with different concentrations of CaS NPs (0, 100, 200, 400, 800, 1000 μM) for 24 hours. After adding 10 μL of Cell Counting Kit-8 (CCK-8, Beyotime, Shanghai, China) to each well, the cells were incubated for an additional 4 hours. Absorbance at 450 nm was measured using a microplate reader, with untreated cells set to 100% viability.

#### OGD/R model

The medium was switched from high-glucose DMEM containing 10% FBS to glucose-free DMEM to simulate ischemia. After incubation in a hypoxic environment (37 °C) for 12 hours, cells were returned to the normoxic incubator, and fresh high-glucose medium was added to simulate reperfusion for 24 hours. The control group consisted of cells maintained in high-glucose DMEM under normoxic conditions. Four experimental groups were prepared: Normal control (Control), CaS NPs-only (200 μM), OGD/R model, and OGD/R + CaS NPs treatment (200 μM).

#### ROS scavenging activity in OGD/R model

Intracellular ROS levels were assessed using dihydroethidium (DHE), which is oxidized by ROS into a fluorescent compound. SH-SY5Y cells were seeded in 24-well plates and subjected to OGD for 12 hours. After incubation in complete medium with different treatments for 24 hours, cells were washed with PBS and incubated with DHE (10 μM) for 30 minutes. Fluorescence images were obtained using a standard fluorescence microscope.

Apoptosis Assay: The anti-apoptotic effect of CaS NPs was evaluated by first exposing SH-SY5Y cells to OGD for 12 hours followed by reperfusion for 24 hours in high-glucose DMEM containing 200 μM CaS NPs. Apoptosis was assessed using Annexin V-PE and 7-AAD staining, followed by flow cytometric analysis on a flow cytometer (Beckman, Indianapolis, USA).

### *In vivo* experiments

#### Animals

Adult male C57BL/6 mice (approximately 25 g, 8 weeks old) were obtained from Guangdong Zhiyuan Biological Medicine Technology Co., Ltd. All animal procedures were approved by the Animal Welfare and Ethics Committee of Guangzhou Medical University (Approval No: P2025-35013), and all experiments adhered to the Guide for the Care and Use of Laboratory Animals.

#### dMCAO model

All male mice were housed under standard specific pathogen-free conditions. Animals were randomly divided into four groups: Sham, Sham + CaS NPs, dMCAO, and dMCAO + CaS NPs. The dMCAO model was established under anesthesia induced with 4%-5% isoflurane and maintained with 1%-2% isoflurane. Following anesthesia, under an operating microscope for precise guidance, the left distal middle cerebral artery was exposed via a subtemporal craniectomy and permanently occluded with bipolar electrocoagulation to induce a focal cortical infarction (20–22). Sham-operated animals underwent the same procedure without occlusion.

#### CaS NPs treatment

Mice received intraperitoneal injections of saline or CaS NPs (3.6 mg/kg) at 30 minutes after dMCAO induction and then at the same time point daily for the following 2 days. Intraperitoneal administration was selected because it allows gradual systemic absorption of nanoparticle formulations and is widely used for preclinical delivery in small animal models. This route can also reduce the potential risk of acute embolic events associated with high−dose intravenous administration of particulate materials while enabling relatively stable systemic exposure. Sham-operated mice receiving CaS NPs (Sham+CaS) were included to evaluate the baseline effects of CaS NPs in the absence of ischemic injury while controlling for the potential physiological influence of anesthesia and surgical manipulation.

#### Neurological function assessment

Neurological deficits were scored 24 hours after treatment as follows: 0, normal motor function; 1, partial failure to extend the right forepaw; 2, circling to the right; 3, falling to the right; and 4, no spontaneous motor activity (21, 23).

Tissue Preparation and Staining: Mice were euthanized by cervical dislocation, and their brains were collected and fixed with 4% paraformaldehyde. After embedding in paraffin, brain sections were stained with Nissl stain and analyzed for neuronal integrity.

#### Immunofluorescence

Immunofluorescence analysis of NeuN (1:300, rabbit; Abcam, USA), GFAP (1:300, mouse; Abcam, USA) and Iba-1 (1:200, rabbit; Abcam, USA) was performed to assess neuronal and glial cell activation, respectively. In addition, TUNEL staining was conducted to evaluate neuronal apoptosis, and DHE staining was used to detect ROS production within the ischemic brain tissue.

#### Western blotting

Brain tissues and cultured cells were lysed using RIPA lysis buffer containing 1 mM phenylmethylsulfonyl fluoride (PMSF) and a protease inhibitor cocktail. The lysates were centrifuged at 12,000 × g for 30 min at 4 °C, and the supernatants were collected. Protein concentrations were determined using a bicinchoninic acid (BCA) protein assay kit (Thermo, USA). Equal amounts of protein were separated by SDS–PAGE and transferred onto polyvinylidene fluoride (PVDF) membranes (Millipore, USA). The membranes were blocked with 5% non−fat milk in TBST for 1 h at room temperature and then incubated overnight at 4 °C with the following primary antibodies: Bax (1:1000, rabbit; Affinity, UK), Bcl−2 (1:1000, rabbit; Affinity, UK), TNF−α (1:500, rabbit; Affinity, UK), IL−1β (1:1000, rabbit; Affinity, UK), and β−actin (1:10000, rabbit; Affinity, UK). After washing with TBST, membranes were incubated with HRP−linked secondary antibodies (1:1000; Yeasen, Shanghai, China) for 1 h at room temperature. Protein bands were visualized using an enhanced chemiluminescence (ECL) detection system and imaged using a Bio−Rad ChemiDoc XRS+ System (Bio−Rad Laboratories, Inc., Hercules, CA, USA). Densitometric analysis of band intensities was performed using Image Lab software (Bio−Rad). The expression levels of target proteins were normalized to the loading control β−actin and expressed relative to the control group. β−actin expression showed no apparent variation across experimental groups under our conditions, supporting its suitability as a loading control for normalization.

#### Detection of H_2_S release

H_2_S levels in the brain were quantified using a H_2_S assay kit (G0133W, Grace Biotechnology, Suzhou, China) after homogenizing tissue samples.

*In Vivo* Biocompatibility Evaluation: On day 3 post-treatment, blood samples were collected for serum biochemistry analysis, including AST, ALT, CR and Urea levels. Major organs were harvested for histological analysis.

### Statistical analysis

#### Image analysis

Brain tissue sections with staining were analyzed using ImageJ to quantify infarct volume and assess changes in immunoreactivity. Protein band densities in Western blot assays were quantified using Image Lab.

#### Statistical analysis

All datasets were first tested for normality using the Shapiro–Wilk test. Data that followed a normal distribution are presented as mean ± standard error of the mean (SEM). Comparisons between two groups were performed using Student’s t−test, while comparisons among multiple groups were analyzed using one−way analysis of variance (ANOVA) followed by Tukey’s *post hoc* test. Data that did not meet the assumption of normality are presented as median ± interquartile range (IQR). For these datasets, differences between two groups were evaluated using the Mann–Whitney U test, and multiple−group comparisons were performed using the Kruskal–Wallis test followed by Dunn’s *post hoc* test. The results of normality testing for all datasets are provided in **Supplementary Table 1**. All statistical analyses were performed using GraphPad Prism version 10 (GraphPad Software, San Diego, CA, USA). A two−tailed P value < 0.05 was considered statistically significant.

## Results

### Donor synthesis and characterization

**Figure 1:**
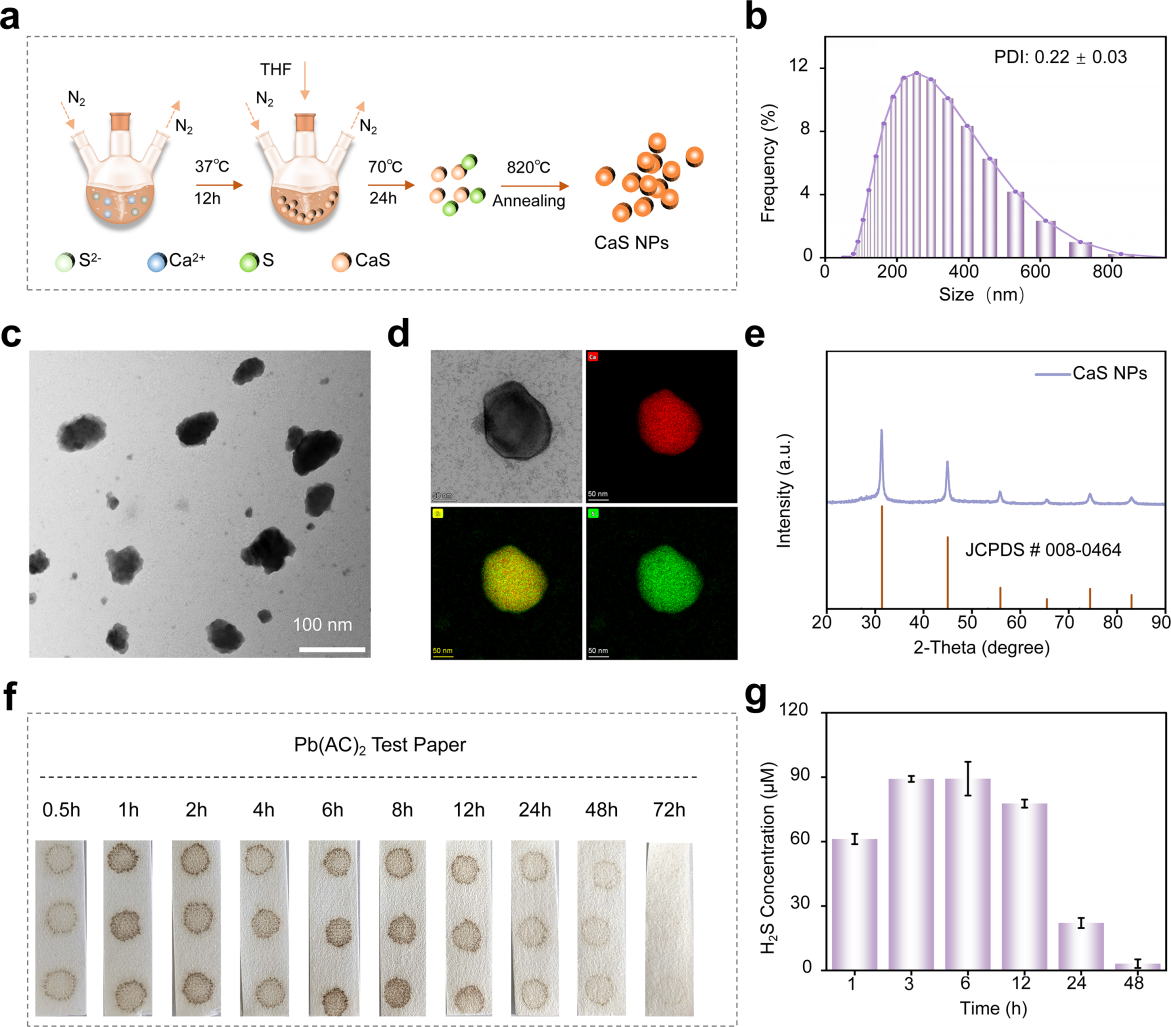
Preparation and characterization of CaS NPs. **(a)** Schematic illustration of the synthesis process of CaS NPs via a wet chemistry method. **(b)** DLS profile of CaS NPs in PBS buffer (pH = 7.4). **(c)** TEM image of CaS NPs. **(d)** TEM image and the corresponding elemental mapping of CaS NPs. **(e)** X-ray diffraction (XRD) pattern of the as-synthesized CaS NPs. **(f)** Color change of a Pb(CH_3_COO)_2_ test paper upon exposure to H_2_S generated from a CaS NPs solution (3000 μM). **(g)** Time-dependent release profile of H_2_S from an aqueous solution of CaS NPs (300 μM). CaS NPs, calcium sulfide nanoparticles.

Conventional H_2_S donors, such as Na2S and NaHS, are highly soluble in aqueous solutions and typically release H_2_S rapidly after dissolution. Based on this rationale, we prepared CaS NPs using a wet-chemistry method (**Figure 1a**) (18, 24). These nanoparticles exhibited low solubility in aqueous environments. Dynamic light scattering (DLS) and transmission electron microscopy (TEM) were used to evaluate the size and morphology of the CaS NPs. The results revealed an average diameter of 228.4 ± 2.6 nm, with a polydispersity index (PDI) of 0.22 ± 0.03 (**Figures 1b-d**). Elemental mapping showed a relatively uniform distribution of calcium and sulfur. X-ray diffraction (XRD) analysis showed that the diffraction pattern of CaS NPs closely matched the standard JCPDS card (No. 08-0464), consistent with the formation of crystalline material (**Figure 1e**). To visualize H_2_S release from CaS NPs, Pb(CH_3_COO)_2_ test paper was employed (25). As shown in **Figure 1f**, the color of the Pb(CH_3_COO)_2_ paper gradually changed from white to black with increasing reaction time, indicating continuous H_2_S generation. Quantitative analysis with an H_2_S detection kit showed that a 300 μM solution of CaS NPs maintained H_2_S concentrations above 22.1 μmol/L for 24 h, gradually decreasing to 3.15 μmol/L after 48 h (**Figure 1g**). Furthermore, as shown in **Supplementary Figure 1**, the conventional donor NaHS exhibited a rapid-release profile, reaching a peak H_2_S concentration of 78.5 μmol/L immediately upon contact with the aqueous solution. This release was short-lived, with the donor signal declining to undetectable levels within 12 h. Compared with NaHS, CaS NPs displayed a more prolonged H_2_S release profile under the tested conditions.

### *In vitro* antioxidant, anti-inflammatory, and anti-apoptotic effects of CaS NPs

**Figure 2:**
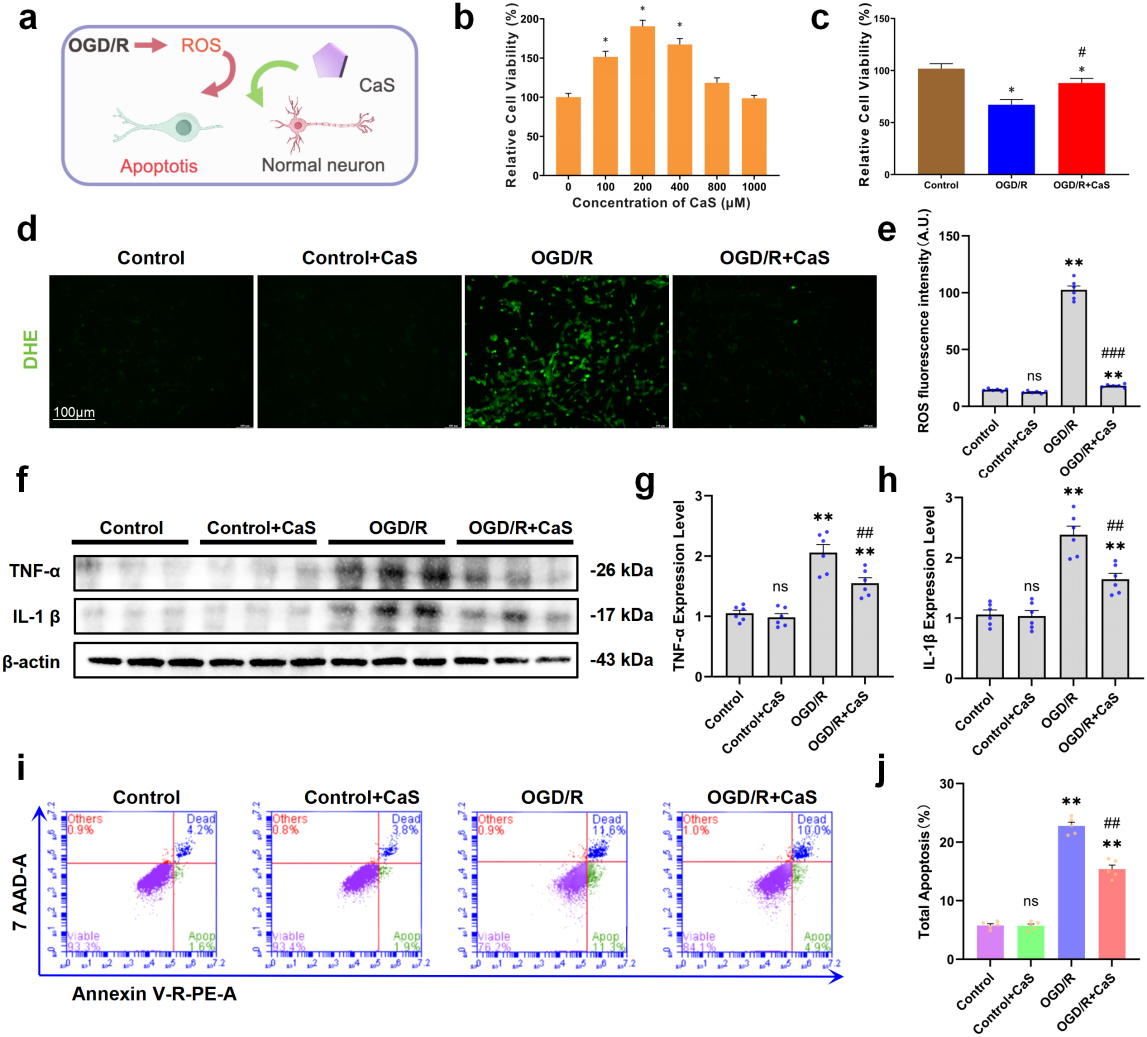
Neuroprotective effects of CaS NPs *in vitro*. **(a)** Schematic illustration of the neuroprotective mechanisms of CaS NPs in neurons subjected to OGD/R injury. **(b)** Viability of SH−SY5Y cells pretreated with different concentrations of CaS NPs for 24 h (n = 5). *p < 0.05. **(c)** Cell viability in different treatment groups after OGD exposure and subsequent reperfusion (R) for 24 h in the presence of CaS NPs (n = 5). *p < 0.05 vs. control; #p < 0.05 vs. OGD/R. **(d)** Representative DHE fluorescence images of SH−SY5Y cells in the OGD/R and other treatment groups. Scale bar: 100 μm. **(e)** Quantitative analysis of ROS fluorescence intensity across treatment groups (n = 6). **p < 0.01 vs. control; ###p < 0.001 vs. OGD/R. **(f)** Expression of TNF−α and IL−1β in SH-SY5Y cells detected by Western blot under different treatments. **(g)** Quantitative analysis of TNF−α expression (n = 6). **p < 0.01 vs. control; ##p < 0.01 vs. OGD/R. **(h)** Quantitative analysis of IL−1β expression (n = 6). **p < 0.01 vs. control; ##p < 0.01 vs. OGD/R. **(i)** Representative flow cytometry plots showing apoptosis in SH−SY5Y cells subjected to OGD/R and incubated with CaS NPs. **(j)** Quantitative results of apoptosis analysis by flow cytometry (n = 6). **p < 0.01 vs. control; ##p < 0.01 vs. OGD/R. Data are expressed as mean ± SEM. Statistical comparisons among groups were performed using one−way ANOVA followed by Tukey’s *post hoc* test. CaS NPs, calcium sulfide nanoparticles.

We next evaluated the effects of CaS NPs *in vitro* and examined several injury-related readouts (**Figure 2a**). The cytocompatibility of CaS NPs was first assessed in SH-SY5Y cells using the CCK-8 assay. As shown in **Figure 2b**, CaS NPs did not induce obvious cytotoxicity across the tested concentration range. At 200 μM, the CCK-8 signal increased to approximately 136%. Even at a higher concentration of 1000 μM, cell viability remained close to 98%, indicating favorable cytocompatibility of CaS NPs under these conditions (11, 26). Even at a higher concentration of 1000 μM, cell viability remained close to 98%, indicating favorable cytocompatibility of CaS NPs under these conditions. Based on these results, 200 μM was selected for subsequent experiments.

To model neuronal ischemia-reperfusion injury *in vitro*, SH-SY5Y cells were subjected to OGD/R. As shown in **Figure 2c**, CaS NPs treatment significantly improved the viability of SH-SY5Y cells following OGD/R injury. Intracellular ROS levels were then measured as an indicator of oxidative stress using the fluorescent probe DHE (**Figure 2d**). Compared with untreated control cells, OGD/R-treated cells exhibited markedly increased fluorescence intensity, indicating elevated ROS production. Treatment with CaS NPs significantly reduced intracellular ROS levels (**Figure 2e**). Inflammatory responses were further evaluated in BV2 cells. In BV2 cells subjected to inflammatory stimulation, CaS NPs treatment markedly decreased the expression levels of IL-1β and TNF-α (**Figures 2f–h**) (27, 28). In BV2 cells subjected to inflammatory stimulation, CaS NPs treatment markedly decreased the expression levels of IL-1β and TNF-α (**Figures 2f–h**). Flow cytometric analysis was further performed to evaluate apoptosis in SH-SY5Y cells after OGD/R injury (**Figure 2i**). The results showed that OGD/R markedly increased the proportion of apoptotic and necrotic cells, whereas treatment with CaS NPs significantly reduced cell death (**Figure 2j**). These findings indicate that CaS NPs attenuated OGD/R-induced neuronal apoptosis under the tested conditions. Collectively, these results indicate that CaS NPs were associated with reduced oxidative stress, inflammatory responses, and neuronal apoptosis *in vitro*.

### Neuroprotective effects of CaS NPs *in vivo*

**Figure 3:**
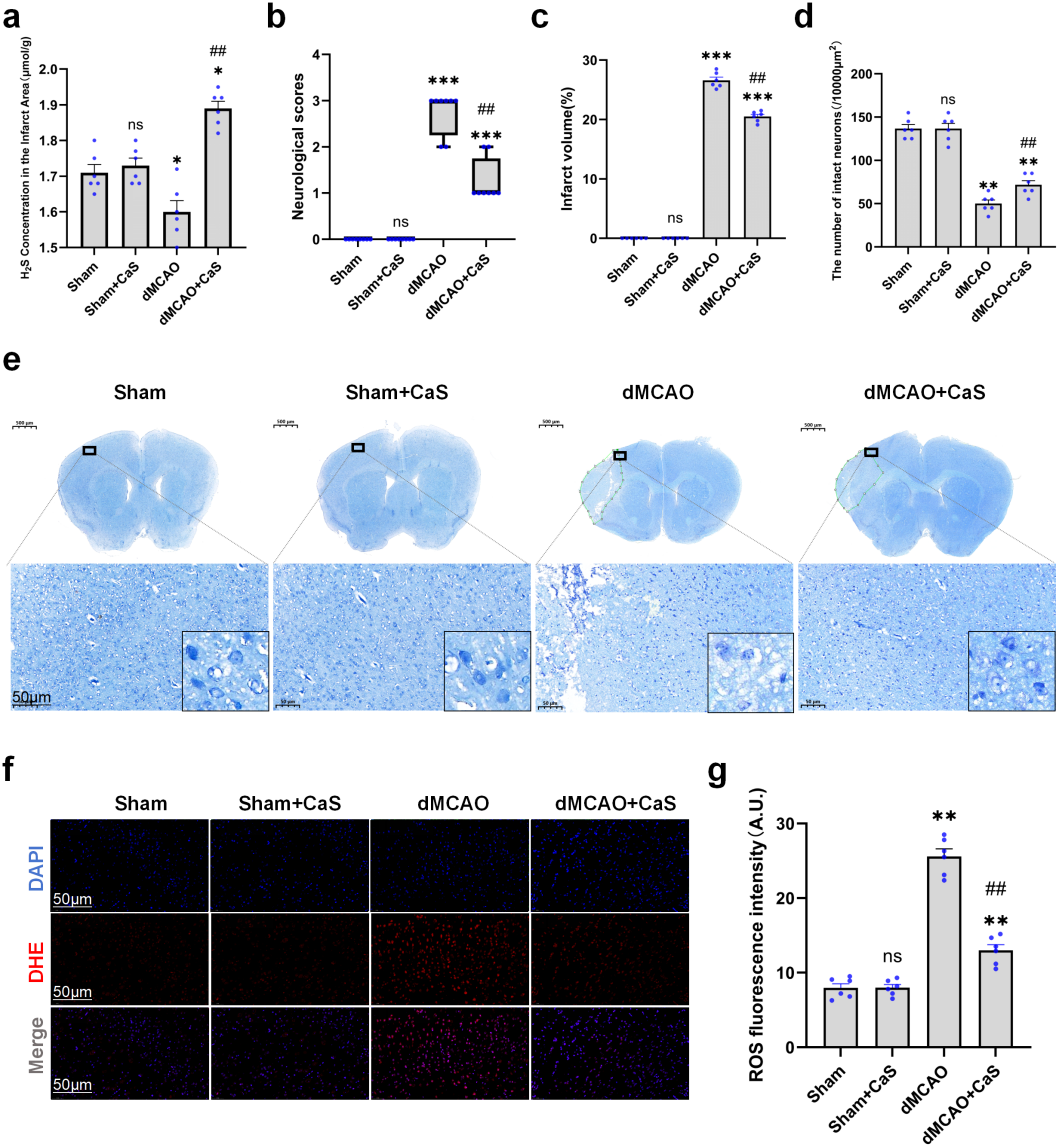
Neuroprotective effects of CaS NPs *in vivo*. **(a)** H_2_S concentration in the cortex (n = 6). *p < 0.05 vs. Sham; ##p < 0.01 vs. dMCAO. **(b)** Neurological function scores of mice in different treatment groups (n = 6). ***p < 0.001 vs. Sham; ##p < 0.01 vs. dMCAO. Data are shown as median ± interquartile range. Statistical comparisons among groups were performed using the Kruskal-Wallis test followed by Dunn’s *post hoc* test. **(c)** Quantitative analysis of cortical infarct volume on day 3 after dMCAO (n = 6). ***p < 0.001 vs. Sham; ##p < 0.01 vs. dMCAO. **(d)** Quantitative analysis of intact neuronal count on day 3 after dMCAO (n = 6). **p < 0.01 vs. Sham; ##p < 0.01 vs. dMCAO. **(e)** Representative Nissl-stained images of brain sections on day 3 after dMCAO. The boxed areas indicate magnified cortical regions. Scale bar: 50 μm. **(f)** Representative DHE fluorescence images showing neuronal ROS levels in the penumbra region of dMCAO mice across treatment groups. Red: DHE; blue: DAPI. Scale bar: 50 μm. **(g)** Quantitative analysis of ROS fluorescence intensity (n = 6). **p < 0.01 vs. Sham; ##p < 0.01 vs. dMCAO. Data are expressed as mean ± SEM. Statistical comparisons among groups were performed using one−way ANOVA followed by Tukey’s *post hoc* test. CaS NPs, calcium sulfide nanoparticles.

The effects of CaS NPs were further evaluated in a dMCAO mouse model induced by electrocoagulation. CaS NPs were administered intraperitoneally 30 minutes after surgery and then once daily at the same time for the following two days. As shown in **Figure 3a**, CaS NPs treatment was associated with elevated H_2_S levels in cortical tissues, consistent with H_2_S release from the nanoparticles. Neurological evaluations were conducted 24 hours after treatment. The CaS NPs-treated group displayed a neurological score of 1.3, whereas the Sham group scored 0 and the model group scored 2.8, indicating partial improvement in the CaS NPs group (**Figure 3b**). By day 3 post-treatment, Nissl staining revealed a reduced infarct size in the CaS NPs group. Specifically, the infarct area in the CaS NPs group was reduced to 20.5%, compared with 26.6% in the model group (**Figure 3c**). Furthermore, the number of intact neurons in the peri-infarct zone was significantly higher in the CaS NPs group than in the model group on day 3 (p < 0.01, **Figure 3d**). There was also a significant decrease in the number of neurons showing cytoplasmic atrophy, morphological changes, and loss of Nissl substance in the infarcted region compared with the model group (**Figure 3e**). To assess oxidative stress in the lesion area, ROS levels were measured using a DHE probe (**Figure 3f**). The data showed that CaS NPs treatment significantly reduced ROS accumulation in the ischemic penumbra compared with the model group (**Figure 3g**). These findings indicate that CaS NPs were associated with reduced neuronal injury and oxidative stress following ischemic stroke.

To provide a preliminary comparison with a conventional H_2_S donor, NaHS was also evaluated in the dMCAO model. Compared with NaHS treatment, CaS NPs showed a trend toward improved neurological outcomes, reduced infarct volume, and better preservation of neuronal structure; however, this comparison was exploratory. The corresponding data are presented in **Supplementary Figures 3**-**S7**.

### Protective effects of CaS NPs on cortical injury following dMCAO

**Figure 4:**
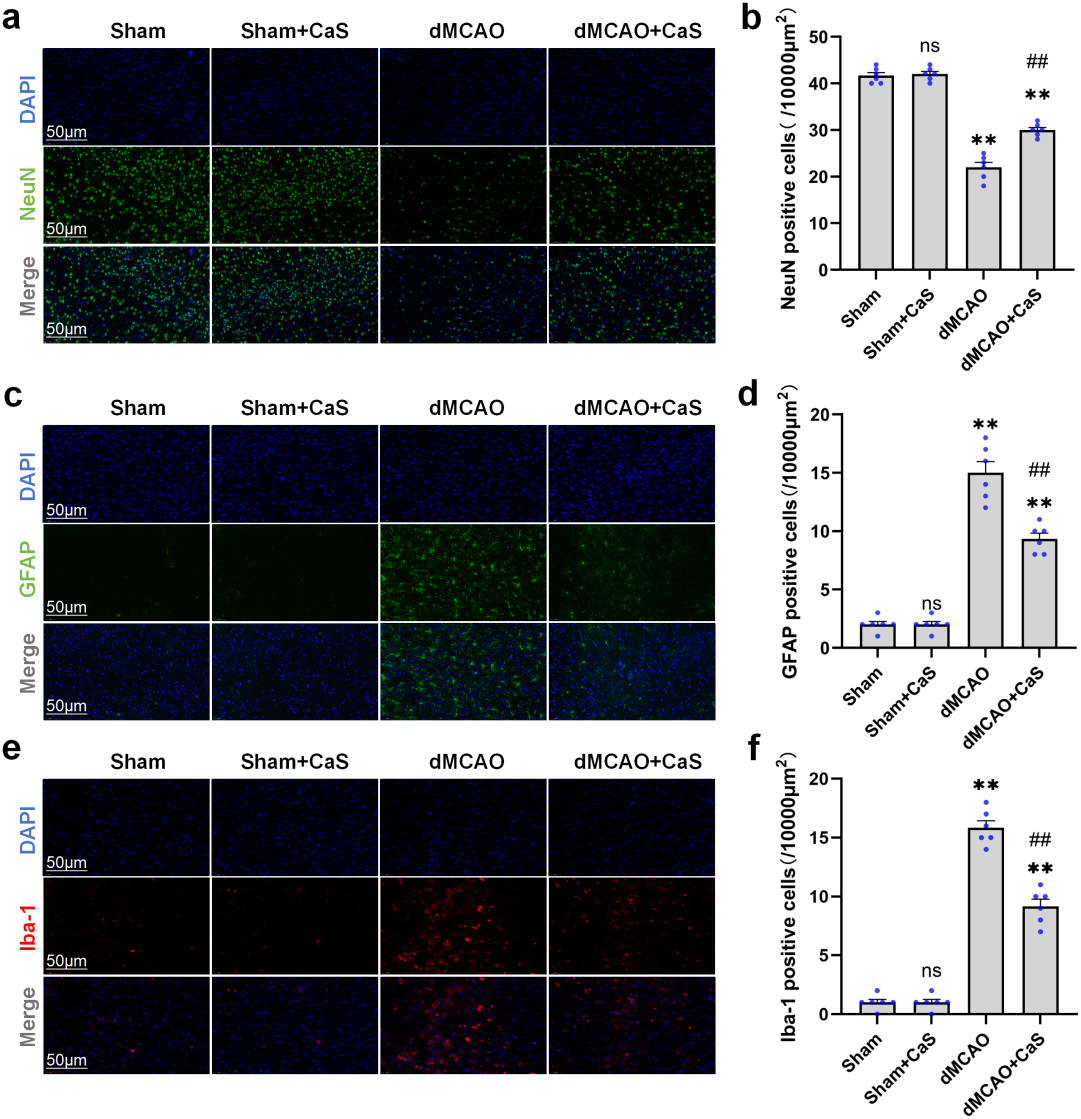
CaS NPs ameliorate cortical injury after dMCAO. **(a, c, e)** Representative images of NeuN-positive neurons, GFAP-positive cells, and Iba-1-positive cells in different treatment groups on day 3 after dMCAO. Scale bar: 50 μm. **(b, d, f)** Quantitative analysis of NeuN-positive neurons, GFAP-positive cells, and Iba-1-positive cells in different treatment groups on day 3 after dMCAO (n = 6). **p < 0.01 vs. Sham; ##p < 0.01 vs. dMCAO. Data are expressed as mean ± SEM. Statistical comparisons among groups were performed using one−way ANOVA followed by Tukey’s *post hoc* test. CaS NPs, calcium sulfide nanoparticles.

As illustrated in **Figure 4a**, three days after dMCAO induction, neurons in the model group exhibited severe damage, including cytoplasmic shrinkage, nuclear deformation, and a pronounced decrease in the NeuN-positive neuronal count. In contrast, animals treated with CaS NPs displayed a higher number of structurally intact neurons at the same time point (**Figure 4b**). Furthermore, the CaS NPs-treated mice exhibited a reduction in the number of GFAP-positive astrocytes and Iba-1-positive microglia compared with the model group (**Figures 4c–f**). These results showed reduced numbers of GFAP-positive astrocytes and Iba-1-positive microglia in the CaS NPs-treated group compared with the model group. Taken together, these results were associated with reduced cortical neuronal injury and glial activation in the dMCAO model.

### Apoptosis- and inflammation-related changes associated with CaS NPs treatment

**Figure 5:**
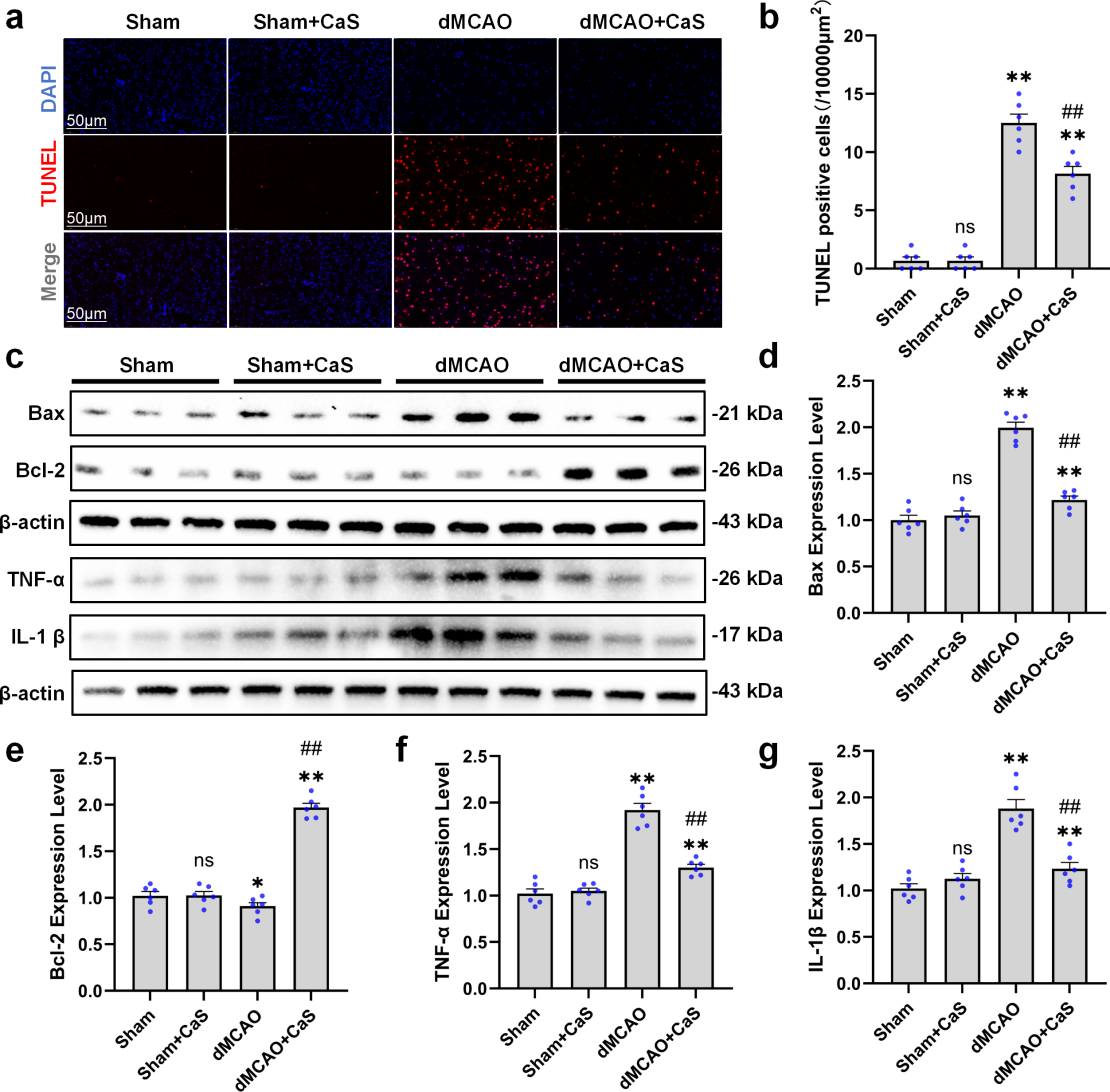
CaS NPs attenuate cortical TUNEL-positive cells and suppress pyroptosis activation after dMCAO. **(a)** Representative images of TUNEL-positive cells in the cortex. Red: TUNEL; blue: DAPI. Scale bar: 50 μm. **(b)** Quantitative analysis of cortical TUNEL-positive cells (n = 6). **p < 0.01 vs. Sham; ##p < 0.01 vs. dMCAO. **(c)** Western blot analysis of Bax, Bcl-2, TNF-α, and IL-1β expression in the cortex after dMCAO. **(d–g)** Quantitative analysis of Bax, Bcl-2, TNF-α, and IL-1β expression levels in the cortex (n = 6). **p < 0.01 vs. Sham; ##p < 0.01 vs. dMCAO. Data are expressed as mean ± SEM. Statistical comparisons among groups were performed using one−way ANOVA followed by Tukey’s *post hoc* test. CaS NPs, calcium sulfide nanoparticles.

We next evaluated apoptosis- and inflammation-related changes associated with CaS NPs treatment. In the model group, the cortex exhibited a marked increase in TUNEL-positive cells compared with the sham group. However, CaS NPs administration significantly decreased the number of TUNEL-positive cells in the cortex three days after dMCAO (**Figures 5a, b**). Western blot analysis further showed that CaS NPs treatment reduced the expression of the pro-apoptotic protein Bax while increasing the level of the anti-apoptotic protein Bcl-2 (**Figures 5c–e**). After three days of treatment, we also examined cortical inflammatory responses. The data indicated that CaS NPs reduced the expression of the pro-inflammatory cytokines TNF-α and IL-1β (**Figures 5c, f, g**). These findings suggest that the observed effects of CaS NPs were associated with reduced apoptosis- and inflammation-related responses.

### *In vitro* and *in vivo* biocompatibility of CaS NPs

**Figure 6:**
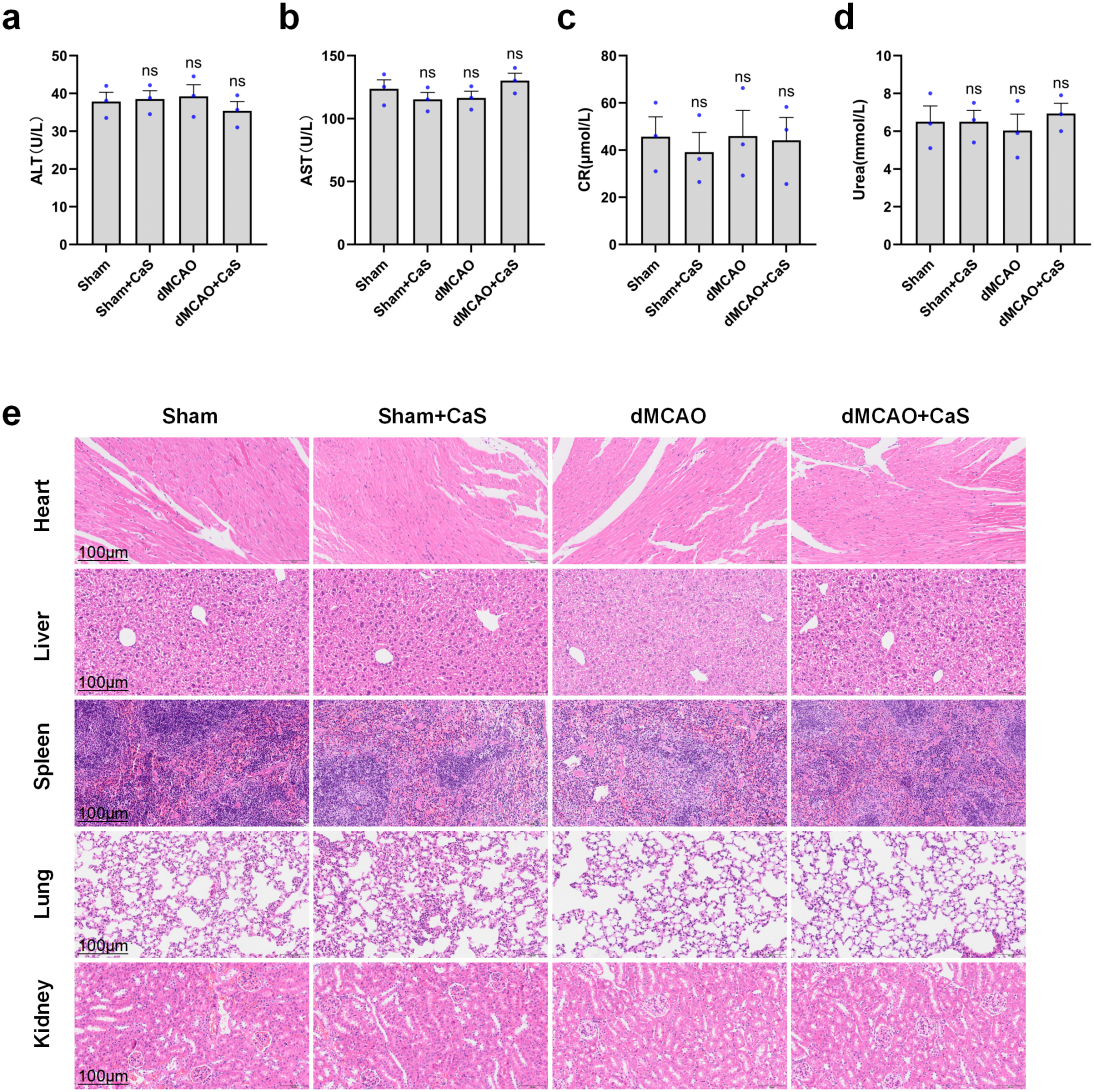
Toxicity profiles of CaS NPs during *in vivo* treatment. **(a–d)** Serum levels of ALT, AST, CR, and Urea in mice following different treatments (n = 3). **(e)** Representative H&E-stained images of major organs from mice in each treatment group. Scale bar: 100 μm. Data are expressed as mean ± SEM. Statistical comparisons among groups were performed using one−way ANOVA followed by Tukey’s *post hoc* test. CaS NPs, calcium sulfide nanoparticles.

CaS NPs did not show significant cytotoxicity at concentrations up to 1000 μM in normal SH-SY5Y cells (**Figure 2b**), indicating favorable cellular compatibility under the tested conditions. In addition, following intraperitoneal administration of CaS NPs for three days, no significant changes were observed in hematological or biochemical parameters in either sham-operated or model mice (**Figures 6a–d**), consistent with a favorable short-term biosafety profile. Histopathological analysis of heart, kidney, liver, lung, and spleen tissues using H&E staining also showed no noticeable changes (**Figure 6e**), further supporting short-term biosafety under the tested conditions.

## Discussion

This study suggests that CaS NPs can function as an intrinsic, carrier-free, slow-releasing H_2_S donor associated with neuroprotective effects across multiple injury-related pathways in experimental ischemic stroke. By leveraging the low solubility and gradual hydrolysis of CaS, we sought to approximate the H_2_S exposure profile to the subacute phase of secondary brain injury and observed oxidative stress-, inflammation-, and apoptosis-related improvements across *in vitro* and *in vivo* models, together with encouraging short-term tolerability. The material-intrinsic “drug depot” nature of CaS avoids the need for additional polymeric carriers, which may simplify formulation design and reduce potential complexities related to pharmacology and biocompatibility (29).

The efficacy and safety of H_2_S are thought to depend substantially on its release kinetics. Fast-releasing inorganic salts (Na2S/NaHS) can generate sharp concentration peaks that may contribute to systemic side effects, whereas organic slow-release donors such as GYY4137 may prolong exposure but often require more complex synthetic procedures or carrier designs and may exhibit batch-dependent release behavior (30–32). Because the primary objective of this study was to evaluate the feasibility of a material-intrinsic slow-releasing H_2_S donor platform, classical rapid-release donors such as NaHS were not included as primary comparators in the main experiments. Consistent with this design concept, CaS NPs maintained detectable H_2_S release over 24–48 h, a timeframe that may be relevant to persistent ROS generation, cytokine signaling, and delayed neuronal death after ischemia-reperfusion (33). In addition, the ischemic microenvironment may facilitate H_2_S release from CaS NPs. Cerebral ischemia is commonly accompanied by metabolic acidosis caused by anaerobic glycolysis and lactate accumulation, resulting in a local decrease in pH (5, 34). Because CaS undergoes hydrolysis in aqueous environments and this process is accelerated under acidic conditions, the ischemic tissue environment may promote greater dissolution of CaS NPs and consequently increased H_2_S generation relative to normal physiological conditions (**Supplementary Figure 2**). Consistent with this release profile, CaS NPs were associated with reduced OGD/R-induced ROS in neurons, lower TNF-α and IL-1β expression in microglia, and decreased neuronal apoptosis *in vitro*. *In vivo*, elevated cortical H_2_S levels, reduced oxidative stress in the ischemic penumbra, and modulation of Bax/Bcl-2 were consistent with the possibility that controlled H_2_S exposure was associated with attenuation of oxidative stress- and apoptosis-related cascades.

Compared with several previously reported donor strategies, CaS NPs may offer a potentially useful balance between release duration and formulation simplicity. Rapid donors mainly provide acute vasodilatory or cytoprotective signaling but may have narrow dosing windows, whereas slow-release organics and encapsulated systems can extend exposure at the cost of greater formulation complexity and potential manufacturing variability (15, 35). Our formulation, characterized by XRD crystallinity and relatively consistent DLS/TEM size distribution, delivered quantifiable H_2_S over up to 48 h and was associated with improvements in behavioral outcomes, infarct burden, oxidative stress-related readouts, and apoptosis-related markers, without obvious organ toxicity on short-term assessment. A preliminary comparison with the conventional fast-releasing H_2_S donor NaHS was also performed in the dMCAO model (**Supplementary Figures 3**-**S7**). Compared with NaHS treatment, CaS NPs showed a trend toward improved neurological recovery, reduced infarct volume, and better preservation of neuronal structure; however, these exploratory findings should be interpreted cautiously because the comparison was preliminary and not designed to establish superiority. Nonetheless, head-to-head benchmarking against leading slow-release donors under identical stroke paradigms remains necessary to establish comparative advantages in efficacy and safety.

These findings provide a rationale for further translational investigation. If a donor’s exposure profile spans the first 48 h after stroke, it may in principle complement reperfusion-based management as an adjunctive neuroprotective approach (36). The improvements in neurological scores and infarct size observed here were moderate in magnitude but may still be relevant to proof-of-concept evaluation. Intraperitoneal delivery served as a proof-of-concept route in mice; future studies should assess clinically relevant routes such as intravenous or intra-arterial administration and determine whether regional exposure can be improved while limiting systemic burden.

We also recognize important limitations. The observation window was largely confined to 72 h, which precludes assessment of long-term recovery, network remodeling, and delayed toxicity. A comprehensive PK/PD framework for H_2_S, including plasma-brain gradients and speciation of sulfane sulfur pools, has not yet been established (37, 38). Whole−body biodistribution, reticuloendothelial uptake, and clearance pathways of the nanoparticles remain to be mapped (39, 40). External validity should also be tested in thromboembolic reperfusion models and in animals with comorbidities such as aging, diabetes, and hypertension. Furthermore, we have not performed direct comparisons with state-of-the-art slow-release donors or evaluated combinations with rt-PA or mechanical thrombectomy. Another limitation is that, although a Sham+CaS group was included to assess the effects of CaS NPs under non-ischemic conditions, a completely untreated CaS-only group without surgical manipulation was not included. While the sham procedure controlled for anesthesia and surgical exposure, the absence of a fully non-stressed drug control group may limit the assessment of baseline systemic safety. In addition, although our results show that CaS NPs elevate cortical H_2_S levels and are associated with reduced oxidative stress-, inflammation-, and apoptosis-related readouts after ischemic stroke, these findings remain primarily correlative. The present study does not directly establish a causal relationship between H_2_S release and the downstream molecular changes observed here. Future studies employing H_2_S scavengers, pharmacological pathway inhibitors, or genetic gain- and loss-of-function approaches will be necessary to further elucidate the underlying signaling mechanisms.

Future work should advance along five lines. First, establish real-time quantification of H_2_S and sulfane sulfur in brain and plasma to define exposure-response relationships, therapeutic index, and peak-to-trough dynamics (41). Second, pursue particle engineering to tune size and surface properties for improved peri-infarct penetration and selectivity, while exploring biodegradable shells or ligand decoration without disrupting intrinsic release, together with stability testing in clinically relevant media and storage conditions (41). Third, validate efficacy in thromboembolic MCAO models with pharmacologic or mechanical reperfusion at clinically relevant delays, and in aged or comorbid cohorts that better reflect patient populations. Fourth, deepen mechanistic resolution around mitochondrial targets, Nrf2 activation, protein persulfidation, and time-resolved state profiling of astrocytes and microglia. Fifth, expand safety and manufacturability studies, including subacute and chronic toxicology, immunogenicity, biodistribution and excretion, and GMP-oriented controls for crystallinity, particle size/PDI, and release rate (42–44).

Taken together, CaS NPs may represent a straightforward platform for slow H_2_S delivery that is compatible with the temporal dynamics of post-ischemic injury. By being associated with reduced oxidative stress-, neuroinflammation-, and apoptosis-related readouts together with a favorable short-term safety profile, CaS NPs may warrant further investigation as an adjunctive neuroprotective strategy. With rigorous PK/PD mapping, particle optimization, and validation in reperfusion and comorbidity models, this strategy may support further preclinical development for acute ischemic stroke.

## Conclusion

CaS NPs were prepared using a wet-chemistry approach and exhibited a relatively sustained H_2_S release profile under the tested conditions. *In vitro*, CaS NPs were associated with reduced oxidative stress- and apoptosis-related readouts in neuronal cells subjected to OGD/R, and reduced the expression of pro-inflammatory cytokines, including TNF-α and IL-1β, in microglial cultures. *In vivo*, CaS NPs were associated with reduced infarct volume in a mouse model of dMCAO, together with improved cortical neuronal preservation and reduced glial activation. Overall, these findings provide proof-of-concept support for further evaluation of CaS NPs as a slow-releasing H_2_S donor in experimental ischemic brain injury.

## Data Availability

The raw data supporting the conclusions of this article will be made available by the authors, without undue reservation.
