# Supplementary material for: Calcium sulfide nanoparticles as sustained H2S donors with neuroprotective potential in ischemic stroke
Source: Front Immunol. 2026 Apr 17;17:1746066. doi: 10.3389/fimmu.2026.1746066 (PMC13132741; doi:10.3389/fimmu.2026.1746066)
Supplement: Supplementary file 1 [file DataSheet1.docx]

**Supplementary Data for**

**Calcium Sulfide Nanoparticles Serve as H_2_S Donors for the Therapy of Ischemic Stroke**

**Zhen Lu^1*^, Yu-Bin Liang^2*^, Zhaotao Wang^3*^, Jianghui Xie^1^, Yiqing Chen^4#^, Yang Li^4,5#^, Zhiqiang Peng^1,2#^**

^1^ Postgraduate cultivation base of Guangzhou University of Chinese Medicine, Panyu Central Hospital, Guangzhou, 510182, China.

^2^ Department of Stroke Center, the Affiliated Panyu Central Hospital of Guangzhou Medical University, Guangzhou 511400, China.

^3^ Department of Neurosurgery, The Second People's Hospital of Fujian Province (The Second Affiliated Hospital of Fujian University of Traditional Chinese Medicine), Fuzhou 350001, China.

^4^ Department of Neurosurgery, Institute of Neuroscience Key Laboratory of Neurogenetics and Channelopathies of Guangdong Province and the Ministry of Education of China The Second Affiliated Hospital of Guangzhou Medical University, Guangzhou 510260, China.

^5^ School of Biomedical Engineering, Guangzhou Medical University, Guangzhou 510006, China.

^#^Correspondence: Zhiqiang Peng, [13926469506@139.com](mailto:13926469506@139.com);

Yiqing Chen^4#^, [yqchen0520@163.com](mailto:wwzztt@126.com); Yang Li^4#^, [lychris@sina.com](mailto:chencm@fjmu.edu.cn).

*These authors contributed equally to this work.

**S1. Time-dependent release profile of H_2_S from an aqueous solution of NaHS**


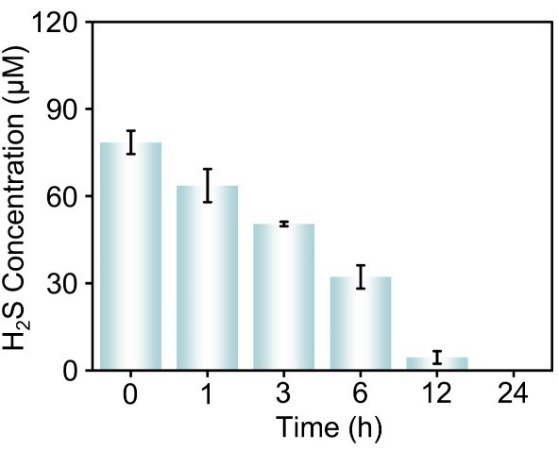


Figure S1. Time-dependent release profile of H_2_S from an aqueous solution of NaHS (300 μM).

**S2. H_2_S concentration of CaS NPs in different pH solutions for various duration**


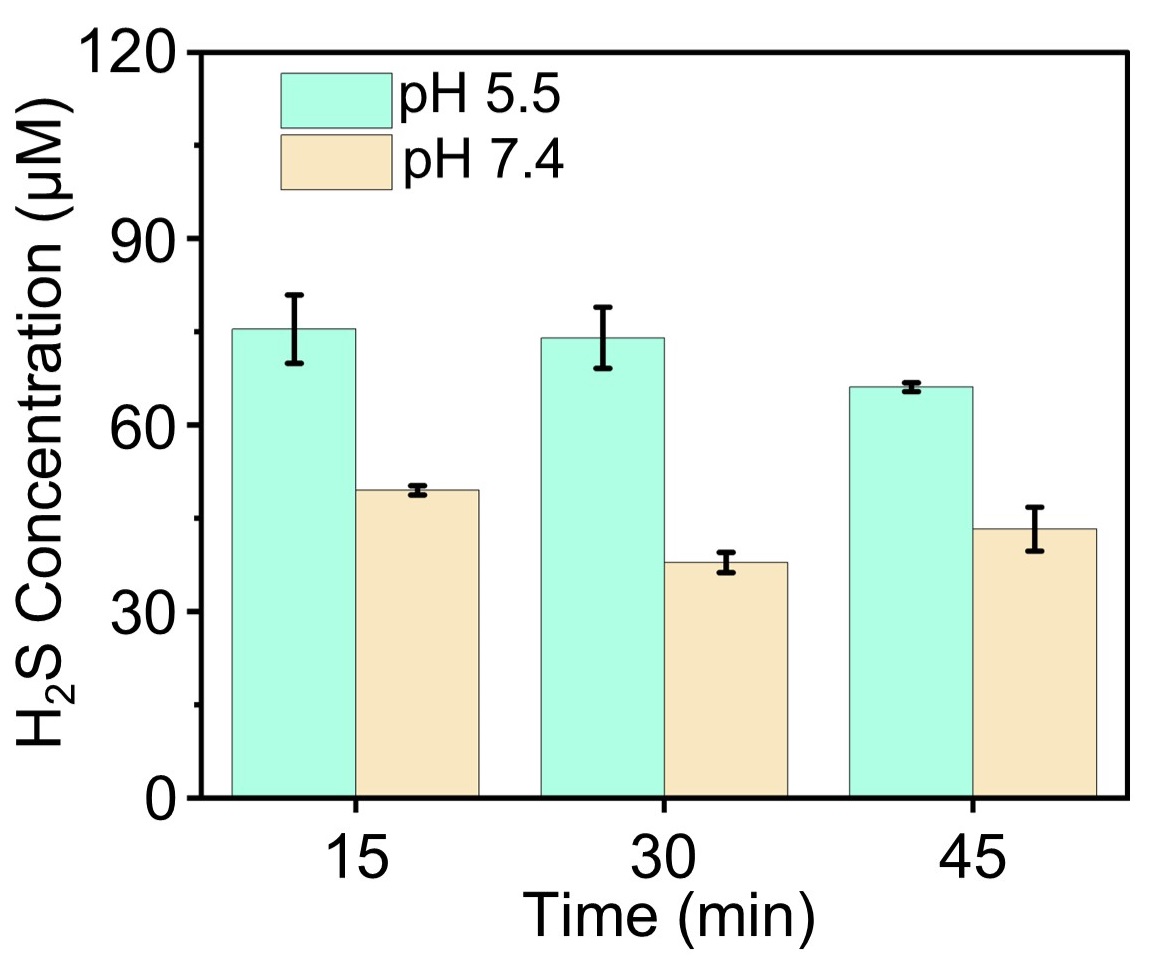


Figure S2. The H_2_S concentration of CaS NPs in different pH solutions for various duration. Abbreviations: CaS NPs, calcium sulfide nanoparticles.

**S3. H_2_S concentration in the cortex**


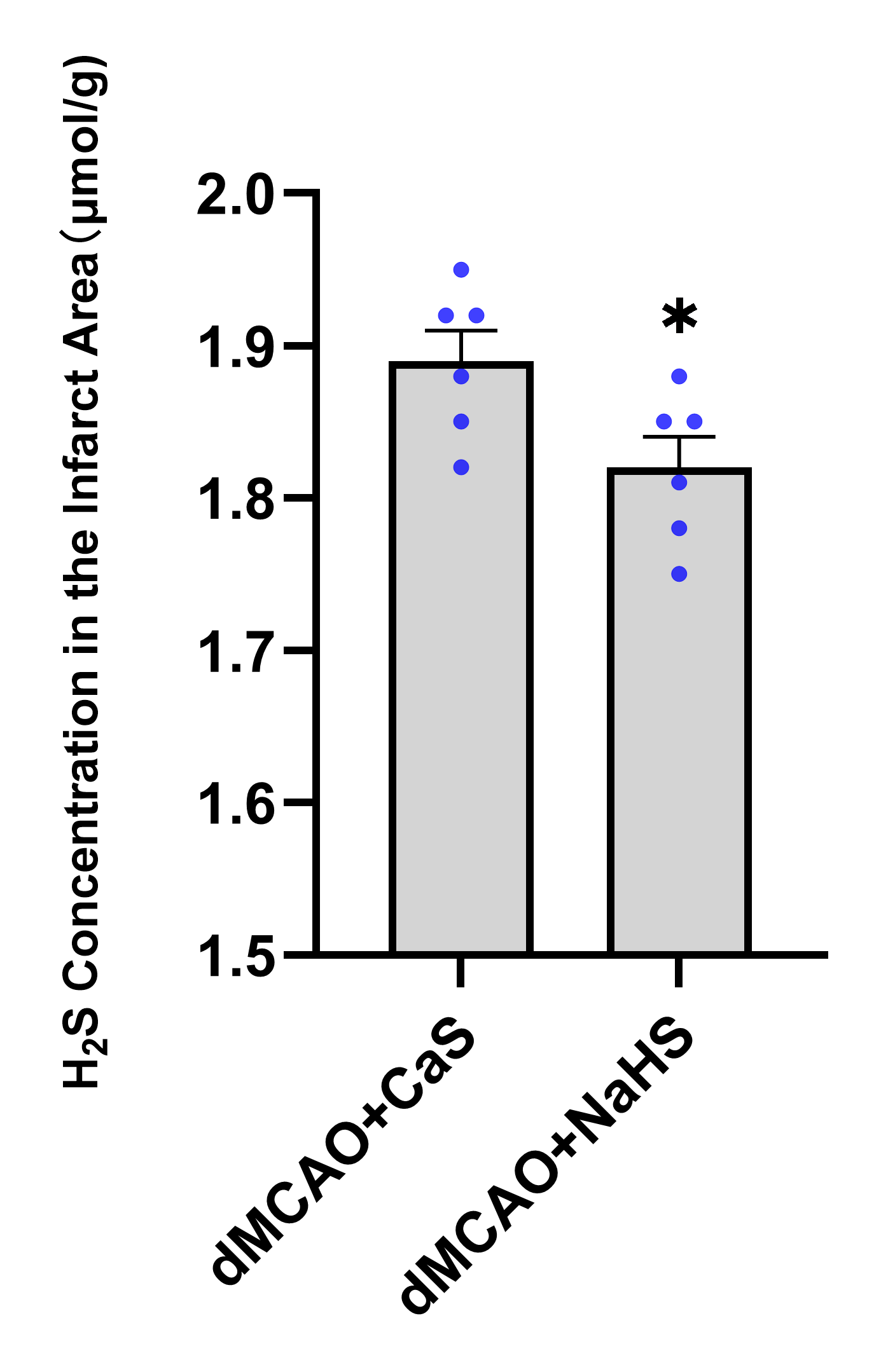


Figure S3. Comparison of cortical H_2_S levels among treatment groups. CaS NPs produced a sustained elevation of H₂S in the ischemic cortex compared with NaHS (n = 6). *p < 0.05 vs. dMCAO+CaS. Data are expressed as mean ± SEM. Statistical comparisons among groups were performed using Student’s t-test. Abbreviations: CaS NPs, calcium sulfide nanoparticles.

**S4. Neurological function scores of mice in different treatment groups**

**
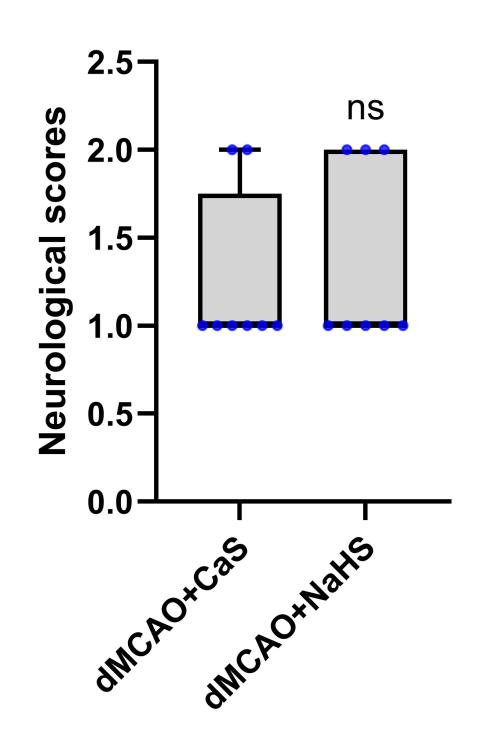
**

Figure S4. Neurological function scores in different treatment groups. CaS NPs–treated mice showed improved neurological outcomes compared with NaHS treatment after dMCAO (n = 6). Data are shown as median ± interquartile range. Statistical comparisons among groups were performed using the Mann-Whitney U test. Abbreviations: CaS NPs, calcium sulfide nanoparticles.

**S5.** **Representative Nissl-stained images of brain sections on day 3 after dMCAO**

**
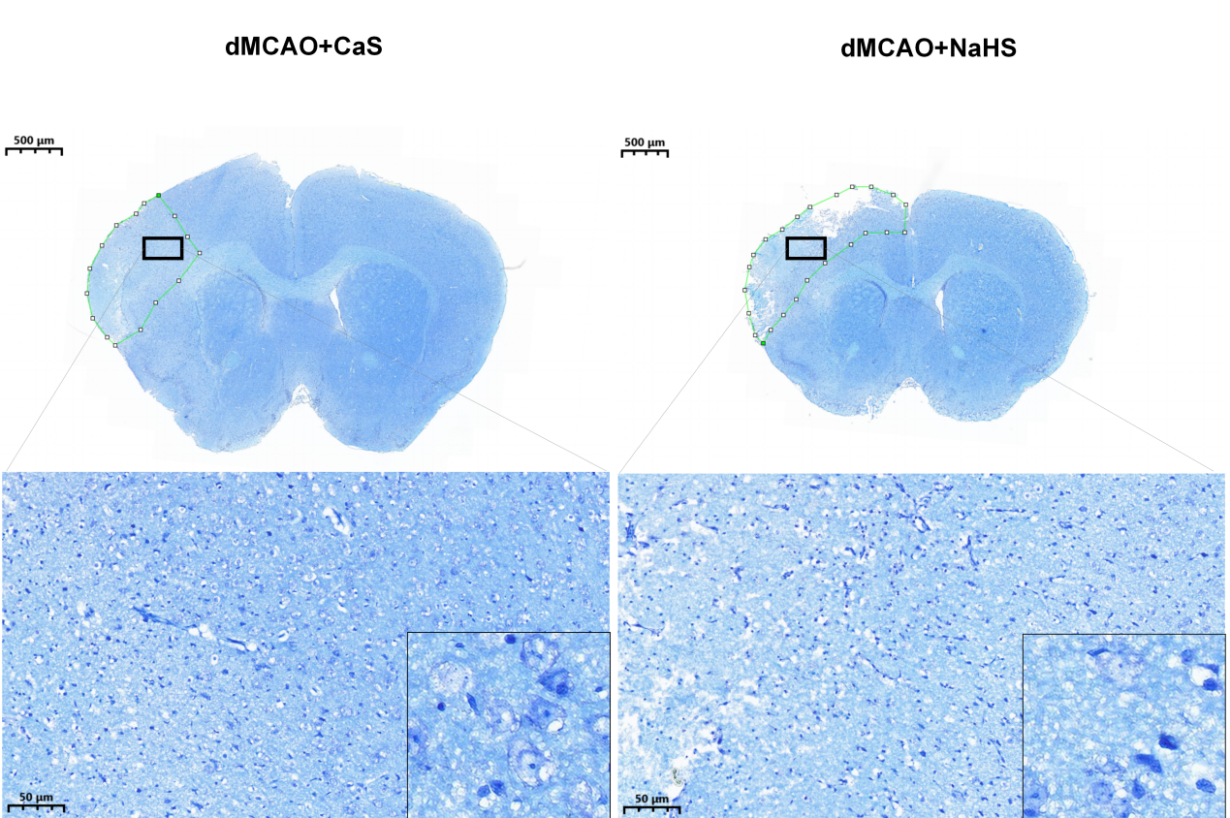
**

Figure S5. Representative Nissl‑stained brain sections on day 3 after dMCAO. CaS NPs treatment preserved neuronal morphology more effectively than NaHS. Abbreviations: CaS NPs, calcium sulfide nanoparticles.

**S6. Quantitative analysis of cortical infarct volume on day 3 after dMCAO**

**
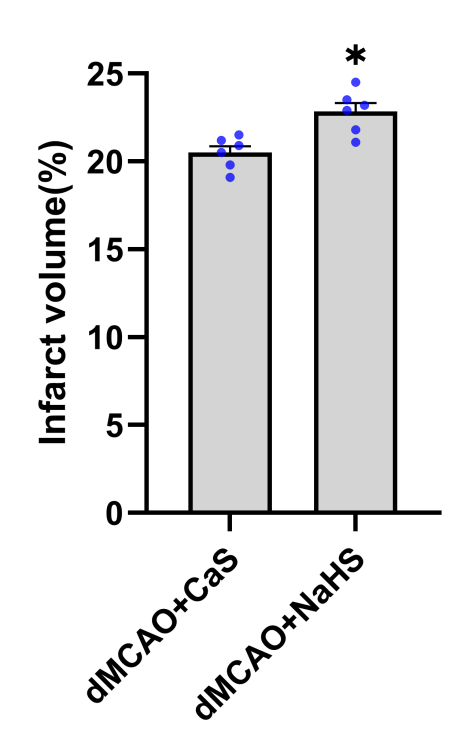
**

Figure S6. Quantitative analysis of cortical infarct volume on day 3 after dMCAO. CaS NPs treatment showed a greater reduction in infarct volume compared with NaHS (n = 6). *p < 0.05 vs. dMCAO+CaS. Data are expressed as mean ± SEM. Statistical comparisons among groups were performed using Student’s t-test. Abbreviations: CaS NPs, calcium sulfide nanoparticles.

**S7. Quantitative analysis of intact neuronal count on day 3 after dMCAO**

**
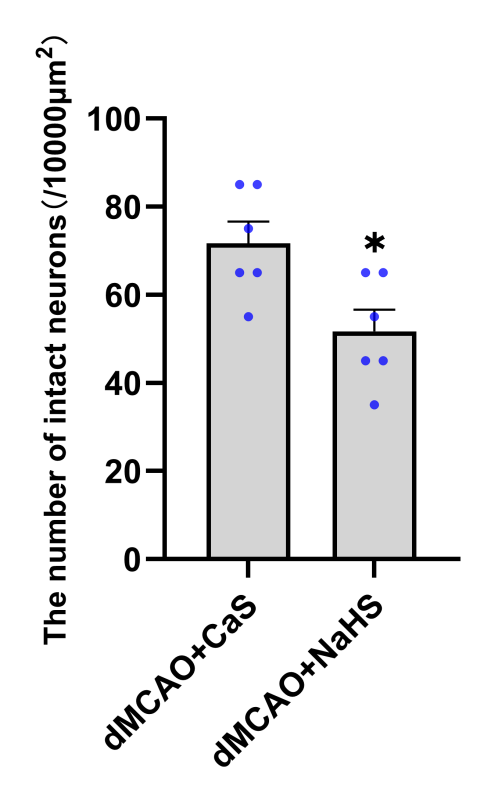
**

Figure S7. Quantitative analysis of intact neuronal counts in the cortex on day 3 after dMCAO. CaS NPs treatment preserved a higher number of intact neurons compared with NaHS (n = 6). *p < 0.05 vs. dMCAO+CaS. Data are expressed as mean ± SEM. Statistical comparisons among groups were performed using Student’s t-test. Abbreviations: CaS NPs, calcium sulfide nanoparticles.

:::
